# The inconsistency between two major aneuploidy-screening platforms—single-nucleotide polymorphism array and next-generation sequencing—in the detection of embryo mosaicism

**DOI:** 10.1186/s12864-022-08294-1

**Published:** 2022-01-18

**Authors:** Dongjia Chen, Yan Xu, Chenhui Ding, Yali Wang, Yu Fu, Bing Cai, Jing Wang, Rong Li, Jing Guo, Jiafu Pan, Yanhong Zeng, Yiping Zhong, Xiaoting Shen, Canquan Zhou

**Affiliations:** 1 Reproductive Medicine Center, The First Affiliated Hospital of Sun Yat-sen University, Guangzhou, 510080 Guangdong China; 2 Guangdong Provincial Key Laboratory of Reproductive Medicine, Guangzhou, Guangdong China; 3 The First Affiliated Hospital of Hainan Medical University, Hainan Medical University, Haikou, Hainan China

**Keywords:** Mosaicism, Preimplantation genetic testing for aneuploidies, Single-nucleotide polymorphism array, Next-generation sequencing, Multiple displacement amplification

## Abstract

**Background:**

In preimplantation genetic testing for aneuploidy (PGT-A), appropriate evaluation of mosaic embryos is important because of the adverse implications of transferring embryos with high-level mosaicism or discarding those with low-level mosaicism. Despite the availability of multiple reliable techniques for PGT-A, data comparing the detection of mosaicism using these techniques are scarce. To address this gap in the literature, we compared the detection ability of the two most commonly used PGT-A platforms, next-generation sequencing (NGS) and the single-nucleotide polymorphism (SNP) array, for mosaic embryos.

**Results:**

We retrospectively reviewed the data of PGT-A or preimplantation genetic testing for chromosomal structural rearrangements (PGT-SR) conducted at our center from January 2018 to October 2020, and selected blastocysts that underwent aneuploidy screening with both an SNP array and NGS. Trophectoderm biopsy, multiple displacement amplification (MDA), and aneuploidy screening with an SNP array were conducted on the enrolled blastocysts. When the SNP array indicated mosaicism, NGS was performed on the corresponding MDA product for verification. Among the 105 blastocysts diagnosed with mosaicism with the SNP array, 80 (76.19%) showed mosaicism in NGS, with complete and partial concordance rates of 47.62% (50/105) and 18.10% (19/105), respectively. The complete discordance rate of the two platforms was 34.29% (36/105). That is, 10.48% (11/105) of the blastocysts were diagnosed with completely different types of mosaicism with the two platforms, while 13.33% (14/105) and 10.48% (11/105) of the embryos diagnosed as showing mosaicism with SNP were detected as showing aneuploidy and euploidy with NGS, respectively.

**Conclusions:**

The consistency of NGS and the SNP array in the diagnosis of embryo mosaicism is extremely low, indicating the need for larger and well-designed studies to determine which platform is more accurate in detecting mosaic embryos.

**Supplementary Information:**

The online version contains supplementary material available at 10.1186/s12864-022-08294-1.

## Background

Mosaicism refers to the phenomenon wherein two or more cell lines with different chromosomes exist in an embryo simultaneously, usually as a result of abnormal chromosomal segregation during mitosis [1]. The incidence of mosaic embryos in the cleavage stage is relatively high, ranging from 15 to 75% [2], while the corresponding incidence is approximately 20–30% in the blastocyst stage [3].

Preimplantation genetic testing for aneuploidies (PGT-A) is an important method for avoiding the transfer of aneuploid embryos and thus reducing the miscarriage rate and shortens the time to achieve live birth. As the third type of embryo, other than euploid and aneuploid embryos, the transfer of mosaic embryos into the uterus as well as the risks associated with transfer have increasingly attracted the attention of clinicians and patients undergoing PGT-A cycles. At present, there is increasing evidence suggesting that mosaic embryos have definite reproductive potential and can result in healthy live births after transfer [4–7]. However, in comparison with euploid embryos, mosaic embryos lead to a higher miscarriage rate and poorer clinical outcomes [8]. Therefore, the accuracy of PGT-A in diagnosing mosaic embryos is very important.

Array-based comparative genomic hybridization (aCGH), single-nucleotide polymorphism (SNP) arrays, and next-generation sequencing (NGS) are the platforms currently used for comprehensive chromosome screening (CCS). In comparison with PGT-A 1.0, which relies on fluorescence in situ hybridization (FISH) and cleavage biopsy, the utilization of PGT-A 2.0, which relies on CCS and trophectoderm biopsy, has been demonstrated to yield significantly better clinical outcomes [9]. Among CCS platforms, NGS has emerged as a research hot spot in the field of PGT-A in recent years. Not only is the resolution of NGS higher than that of aCGH and SNP [3], but better clinical results can be obtained by applying PGT-A based on NGS [10, 11]. At present, the gold standard for detecting mosaic embryos has not been accurately described. However, the Preimplantation Genetic Diagnosis International Society (PGDIS) suggests that since only the NGS can measure chromosome copy number, a validated NGS platform should be the only diagnostic platform used to detect mosaicism in a biopsy sample [12]. Importantly, the ability of NGS to detect mosaic embryos has been verified in previous studies by comparing results obtained with NGS to those obtained with FISH, quantitative polymerase chain reaction (qPCR), and aCGH in the same mosaic samples, showing satisfactory accuracy and sensitivity [13–15]. However, few studies have attempted to explore which method (NGS versus an SNP array) provides more accurate detection of mosaic embryos. To this end, we retrospectively analyzed the data of embryos detected as showing mosaicism with an SNP array platform and subsequently verified them using an NGS platform. We expected to evaluate the consistency of NGS and the SNP array in detecting mosaic embryos and provide reference data for the clinical selection of PGT-A detection platforms.

## Results

A total of 88 couples were included in this study. The mean age of the female participants was 31.2 ± 4.5 years, and the mean age of the male participants was 33.1 ± 4.9 years (Table 1). Twenty-five couples (28.4%) received PGT-A treatment: 23 due to recurrent miscarriage, advanced age, or repeated implantation failures, and two due to maternal whole-chromosome aneuploidy mosaicism (45,XO[88]/47,XXX, [12]; 47,XX + 8 [19]/46,XX, [11]). The remaining 63 couples (71.6%) received preimplantation genetic testing for chromosomal structural rearrangements (PGT-SR) treatment: 58 due to chromosome balanced translocation, one due to maternal mosaicism for chromosome balanced translocation (46,XX,t(2;11)(p21;q23.3) [8]/46,XX [22]), one due to chromosome inversion, and three due to combined chromosome balance translocation and inversion.

**Table 1:** Basic characteristics of the 88 couples involved

Basic characteristics	Mean ± SD or n (%)
Female age (years)	31.02 ± 4.47
Male age (years)	32.80 ± 4.65
PGT-A	25 (28.4%)
PGT-SR	63 (71.6%)
Indication	
RM/ AMA/ RIF	23 (26.1%)
Maternal whole chromosome aneuploidy mosaicism	2 (2.3%)
Balanced translocation	59 (69.3%)
Chromosome inversion	1 (1.1%)
Balanced translocation and chromosome inversion	3(1.1%)

*PGT-A* preimplantation genetic testing for aneuploidy, *PGT-SR* preimplantation genetic testing for chromosomal structural rearrangements, *RM* recurrent miscarriage, *AMA* advanced maternal age, *RIF* recurrent implantation failure

A total of 105 blastocysts were judged as showing mosaicism on using the SNP array. Among them, 49 (46.67%) embryos showed whole-chromosome aneuploidy mosaicism, 55 (52.38%) showed segmental chromosome aneuploidy mosaicism, and one (0.95%) embryo showed both segmental chromosome aneuploidy mosaicism and whole-chromosome aneuploidy mosaicism.

**Fig. 1:**
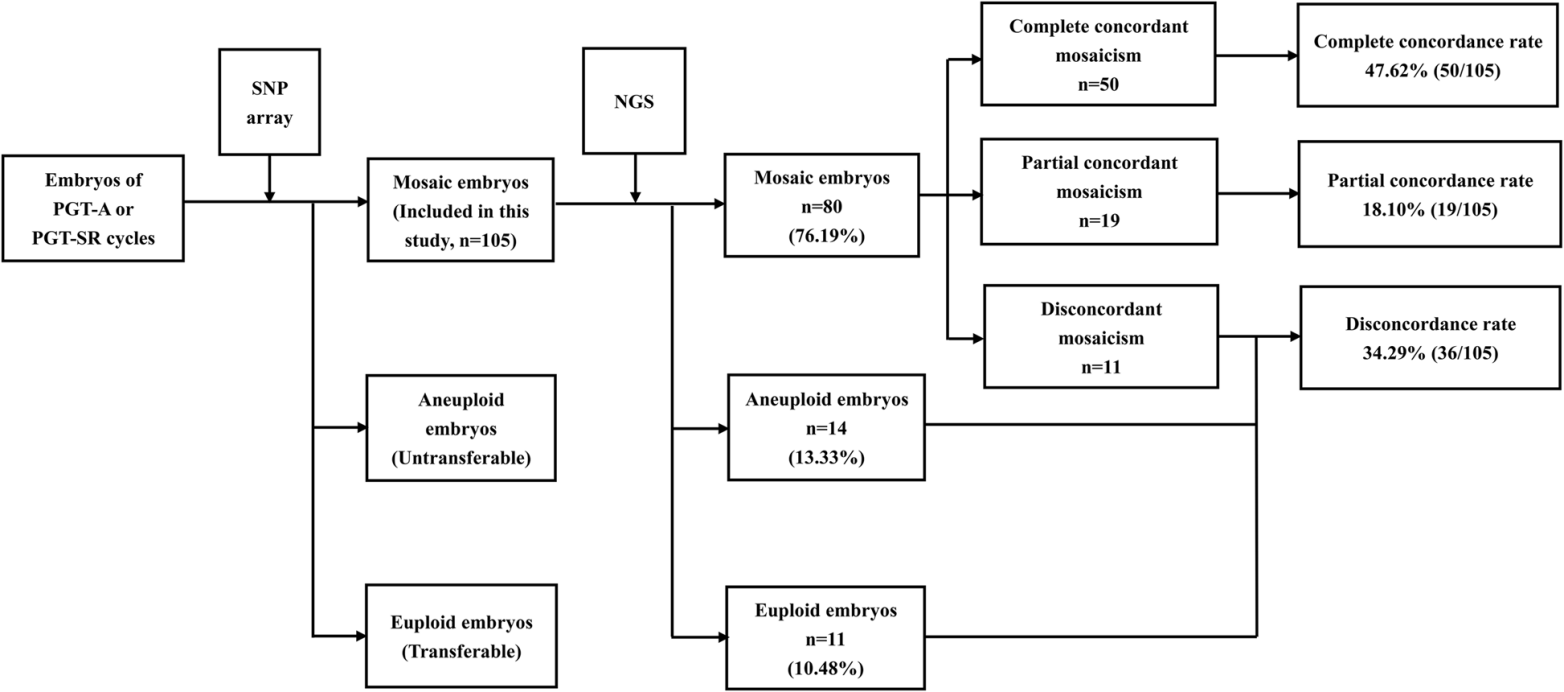
Flowchart of methods and main results of this study

**Fig. 2:**
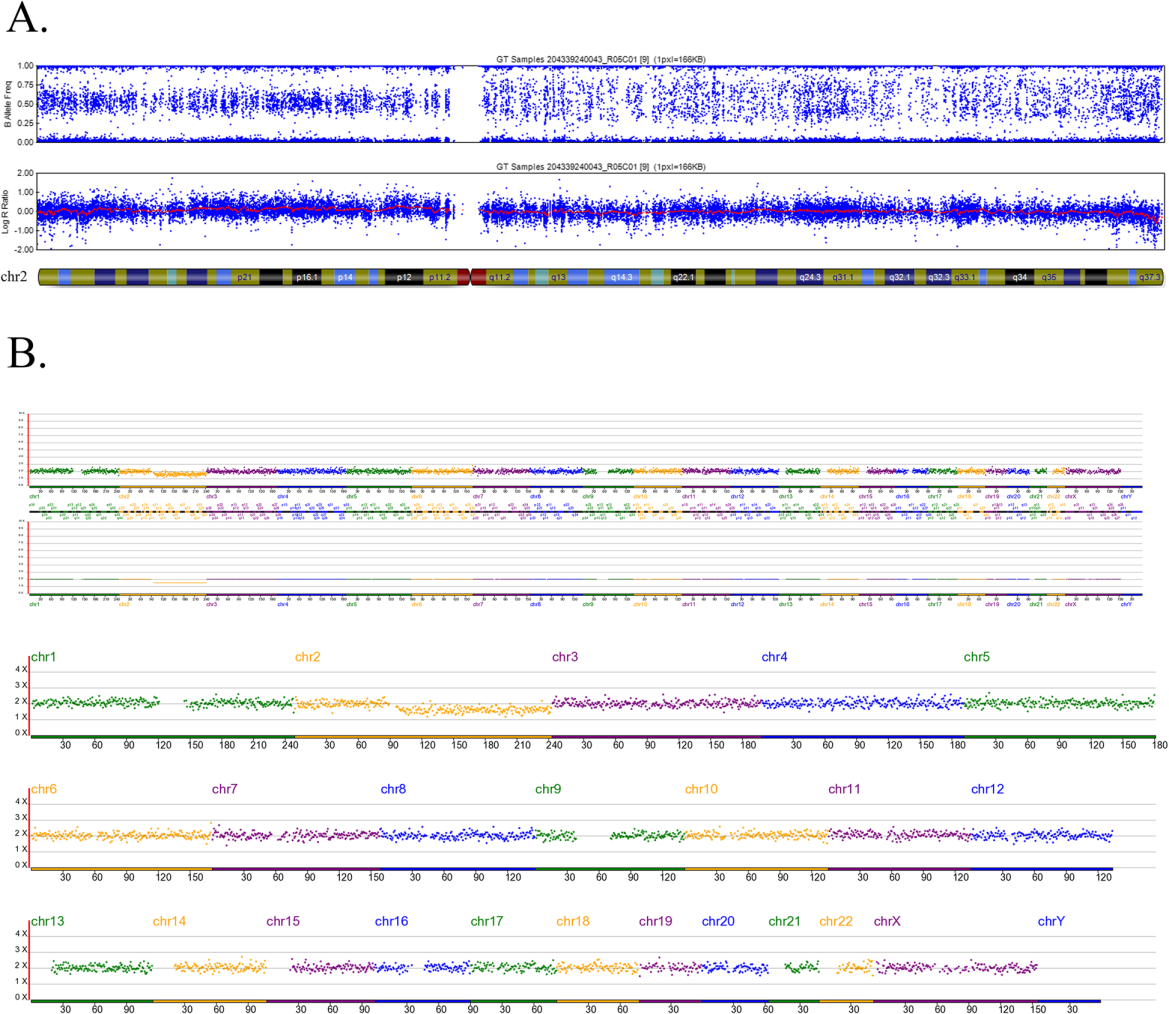
An example of a consistent result obtained by SNP array and NGS. This blastocyst (No.6 blastocyst in Additional file 1) was diagnosed with -mos(2q) using both SNP array (**A**) and NGS (**B**)

**Fig. 3:**
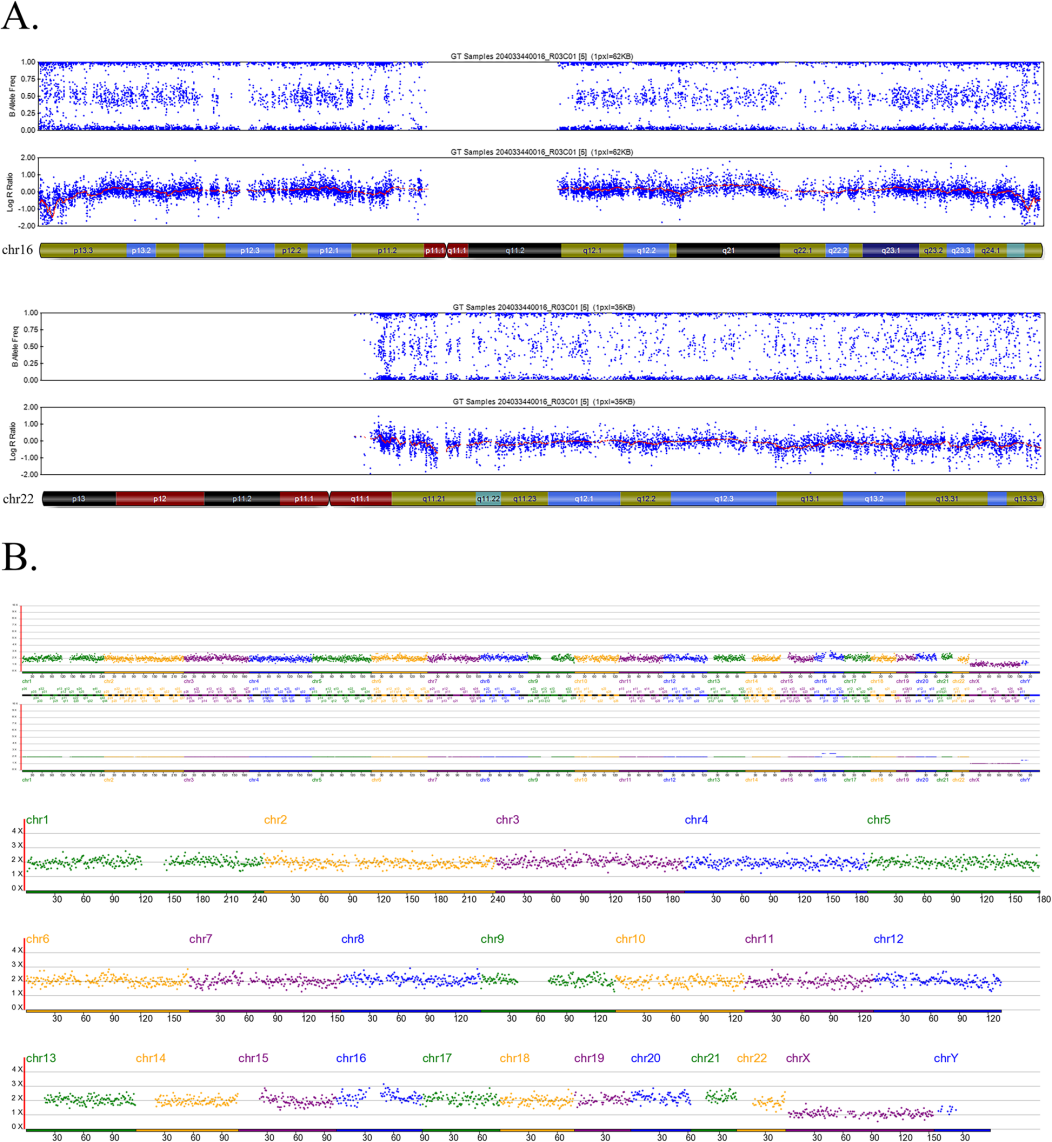
An example of inconsistent results obtained by SNP array and NGS. This blastocyst (No.71 blastocyst in Additional file 1) was diagnosed with -mos(22) using SNP Array (**A**). However, NGS reported no chromosomal abnormality on chromosome 22 but a segmental duplication mosaicism on chromosome 16 (p12.2-q21) (**B**)

**Fig. 4:**
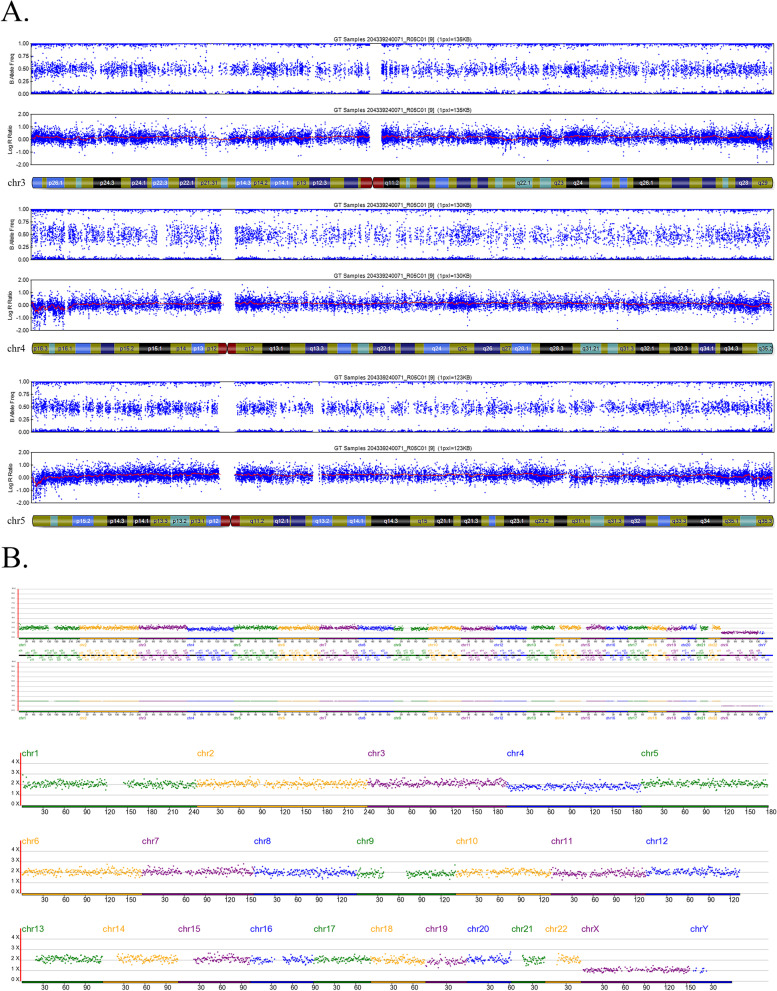
Another example of inconsistent results obtained by SNP array and NGS. This blastocyst (No.82 blastocyst in Additional file 1) was diagnosed with +mos(4) using SNP Array (**A**). However, NGS reported that it was euploid (**B**)

Since the SNP array cannot report the mosaic level of the embryos, a separate aliquot of the multiple displacement amplification (MDA) products of the alleged mosaic embryos was sampled and tested using the NGS platform. Clear NGS diagnoses were obtained for 100% (105/105) of the embryos, and NGS evaluation diagnosed mosaicism in 80 of these embryos (76.19%; Fig. 1, Table 3), with complete concordance, partial concordance, and discordance rates of 47.62% (50/105), 18.01% (19/105), and 10.48% (11/105), respectively. Moreover, NGS identified aneuploidy and euploid status in 14 (13.33%) and 11 (10.48%) embryos, respectively. Thus, the overall discordance rate between the two detection platforms was 34.29% (36/105). Figures 2, 3 and 4 show examples of consistent and inconsistent results obtained by SNP arrays and NGS.

To explore the potential influencing factors of the consistency between the two platforms in mosaicism detection, we stratified 105 embryos according to the whole chromosome mosaicism or segmental mosaicism, maternal age, and embryo quality, and compared them to determine whether there were differences in complete consistency between the groups (Table 2). The complete concordance rate of the two methods was similar between the whole-chromosome aneuploid mosaic embryos (*n* = 49) and segmental chromosome aneuploid mosaic embryos (*n* = 55) determined using SNP (42.86% vs 52.73%, *P* = 0.315) (Table 2). After grouping the cases according to female age < 35 years (*n* = 82) and ≥ 35 years (*n* = 23), the complete concordance rate of the two methods was still similar (45.12% vs. 56.52%, *P* = 0.333) (Table 2). For high-quality (*n* = 80) and low-quality (*n* = 25) blastocysts, the complete concordance rate of the two methods were 47.50 and 48.00%, respectively, and the difference was still not statistically significant (*P* = 0.965) (Table 2).

**Table 2:** Concordance rate of NGS and the SNP array in stratified analysis

	Concordance rate	***P*** value
Total	50/105 (47.62%)	
Female age		
< 35 years	37/82 (45.12%)	0.333
≥ 35 years	13/23 (56.52%)	
Type of aneuploidy ^a^		
Whole chromosomal aneuploidy	21/49 (42.86%)	0.315
Segmental aneuploidy	29/55 (52.73%)	
Good-quality embryo		
Yes	38/80 (47.50%)	0.965
No	12/25 (48.00%)	

^a^ One embryo was excluded from this analysis because it was reported by SNP array with both whole-chromosome aneuploidy mosaicism and segmental chromosome aneuploidy mosaicism

Among the 80 embryos diagnosed as showing mosaicism using NGS, 71.25% (57/80) had a mosaicism level ≤ 50, and 28.75% (23/80) had a mosaicism level > 50%. Thus, the proportion of transferable embryos (euploid embryos or embryos with ≤50% chromosome mosaicism) was 64.76% (68/105), and the proportion of untransferable embryos (aneuploid embryos or embryos with > 50% chromosome mosaicism) was 35.24% (37/105) (Table 3).

**Table 3:** Summary of the NGS-PGT-A and FET results for the 105 alleged mosaic blastocysts diagnosed by SNP array

NGS-PGT-A and FET results	n (%)
NGS-PGT-A outcomes (*n* = 105)	
Euploid embryo	11 (10.48%)
Aneuploid embryo	14 (13.33%)
Mosaic embryo	80/105 (76.19%)
Mosaic embryo with low aneuploid percentage (≤50%)	57/80 (71.25%)
Mosaic embryo with high aneuploid percentage (> 50%)	23/80 (28.75%)
Transferable embryo	68/105 (64.76%)
Untransferable embryo	37/105 (35.24%)
FET outcomes (*n* = 21) ^a^	
Not pregnant	5
Biochemical pregnancy	15
Clinical pregnancy	14
Early miscarriage	2
Ongoing pregnancy (under observation)	7
Live birth	5

^a^ The incidence of each pregnancy outcome was not calculated because the FET outcomes of some embryos were under observation

Of the 68 transplantable embryos, 21 (5 euploid embryos and 16 embryos with < 50% mosaicism determined using NGS) were transferred back to the uterus in subsequent frozen-thawed embryo transfer (FET) cycles (Table 3). Of these, 6 did not result in pregnancy after the transfer, and 5 have now reached live births. Two resulted in early miscarriage, seven are in an ongoing pregnancy, and one was recently confirmed to be HCG positive. Unfortunately, no prenatal diagnosis or aneuploidy test was performed in the patients with live births or miscarriages, respectively.

## Discussion

For patients who undergo the PGT cycle for assisted reproduction, every embryo is precious. Thus, it is very important to ensure the accuracy of PGT diagnosis and avoid wastage of embryos with reproductive potential. The clinical significance of embryos diagnosed as mosaic using PGT-A is currently unclear. In 2015, Greco et al. reported for the first time that transferring mosaic embryos could result in live births [5], and this phenomenon has been confirmed in subsequent studies [16]. Recent studies have reported that mosaic embryos can develop into healthy babies with a completely normal karyotype [6, 7], which supports the ability of mosaic embryos to show a certain degree of self-correction [17]. However, in comparison with the outcomes of euploid embryo transfer, the implantation rate of mosaic embryos transfer was significantly lower (30.1 vs 55.8%) and the miscarriage rate was significantly higher (55.6% vs 17.2%) [18]. In a multi-center prospective cohort study based on 137 mosaic and 476 euploid embryos, mosaic embryo transfers led to a significantly lower clinical pregnancy rate (40.1% vs. 59.0%), lower ongoing rate (27.1% vs. 47.0%), and higher miscarriage rate (33.3% vs. 20.5%) than euploid transfers [19].

Zore et al. observed a significant decrease in live birth rates for mosaic embryo transfer (30.0% vs. 53.8%) [19]. Another study showed that among patients who had no euploid embryos, those who chose to restart an additional PGT-A cycle had a significantly higher rate of ongoing pregnancy than those who chose to transfer mosaic embryos (51.2% vs. 27.6%) [20]. Due to the undeniable implantation potential and unsatisfactory transfer outcomes of mosaic embryos, accurate detection and management of these embryos has become a major challenge for PGT-A.

NGS, also known as high-throughput sequencing, has the characteristics of high output, high sensitivity, and high automation. Current research shows that NGS, aCGH, and SNP arrays have a high concordance rate (> 99%) in screening aneuploid embryos [21, 22]. However, only a few studies have compared the effectiveness of these different platforms in the diagnosis of mosaic embryos. The limited current evidence suggests that the sensitivity of NGS for detection of mosaicism is higher than that of aCGH and the SNP array. aCGH and the SNP array are only reliable when the proportion of aneuploid cells in mosaic embryos is 40–60%, and for NGS, the proportion can be as low as 20% [1, 17]. For example, Ruttanajit et al. used aCGH and NGS to evaluate 49 blastocysts, of which six blastocysts (12.2%) were diagnosed inconsistently due to the failure of aCGH to detect mosaicism with an abnormal cell ratio below 30% [15]. Friedenthal et al. observed 548 and 368 PGT-A-FET cycles based on NGS and aCGH, respectively, and found that NGS can exclude more mosaic embryos, embryos with segmental aneuploidy, and trisomy embryos as well as yield better pregnancy outcomes [10]. Furthermore, Niu et al. compared the clinical outcomes of 805 NGS-PGT-A cycles and 613 SNP array-PGT-A cycles, and showed that the clinical pregnancy rate and healthy live birth rate in the NGS-PGT-A group were significantly higher [11]. Moreover, the rate of miscarriage was lower in the NGS-PGT-A group, presumably because NGS can exclude more mosaic embryos [11]. Consistent with this, a recent study based on 6427 blastocysts showed that NGS yielded a significantly higher mosaicism detection rate (23.3% vs 7.7%), and lower spontaneous abortion rate (6.33% vs 10.07%) than the SNP array in PGT cycles [23].

In addition, NGS offers the advantage of detecting the level of mosaicism. Previous studies have verified that NGS can be used to diagnose mosaicism reliably up to 20–80% through a series of cell mixing experiments [4, 18]. A study by Spinella et al. in 2018 showed that the clinical pregnancy rate (15.2% vs. 46.4%), implantation rate (24.4% vs. 54.6%), and live birth rate (15.2% vs. 46.6%) of high-level mosaic embryos (≥50% mosaicism) were significantly lower than those of euploid embryos. In contrast, low-level mosaic embryos (< 50% mosaicism) had similar clinical outcomes as euploid embryos [24]. For this reason, the 2019 PGDIS position statement on mosaic embryo transfer stated that low-level mosaic embryo transfer has priority over high-level mosaic embryos [25]. However, the SNP array, which is the PGT-A platform routinely used by our center, cannot determine the level of mosaicism in embryos. Therefore, in clinical practice in this center, we first use SNP array to perform aneuploidy screening of embryos. For an embryo diagnosed with SNP array, we will further use NGS to verify the diagnosis of mosaicism and obtain the percentage of mosaicism to determine whether they are transferable.

In this study, 105 embryo biopsy samples that were identified as showing mosaicism on the SNP array platform were tested using NGS, and the two platforms showed a large discordance rate (34.29%), with some of these embryos identified as aneuploid (13.33%) and euploid (10.48%) using NGS. Our study suggests an inconsistency between NGS and SNP array in the detection of embryo mosaicism. It is necessary to answer the possible causes of this inconsistency and how to solve this problem. In 2018, Li et al. compared the SNP array and NGS data of 254 human DNA samples, from the Hapmap Project and 1000 Genomes Project, respectively [26]. They found that there was a low consistency (< 30%) between the two platforms for the detection of genome-wide copy number loss. They then used a precise algorithm, CNVhac, to detect copy number losses directly from the HapMap microarray data and found that 88% of these copy number losses were supported by breakpoint sequences in the NGS raw data, suggesting the importance of bioinformatics algorithms in the consistency inference between the two platforms. It is noteworthy that our study only used one type of NGS Pipelines. However, a diversity of Software/Pipes has been developed for the CNV detection in NGS Data, but these often yield contradictions on the same dataset [27–30]. Attempting to use multiple algorithms for each platform or develop more accurate algorithms to get more consistent detection results are worthwhile. Moreover, it would be interesting to see if the discordancy could be explained through quality control (QC) metrics. Unfortunately, because the analysis methods of these two platforms are very different, and because our center does not have more powerful bioinformatics analysis ability to support such research, we could not explain the source of inconsistency through the QC metrics of the two platforms. However, this is a very significant question, which deserves further exploration in future research.

On the other hand, there is room for improvement in both platforms to help narrow the differences further. For SNP array, due to the low quality of SNP data and insufficient training of segmentation algorithm, fragments or breakpoints detected using CNV detection software of SNP array often need manual review to avoid false positives. However, the graphical representation of the results of the SNP array are not as good as those of the NGS platform, which causes some problems for users. To this end, optimizing the visualization of SNP array data to more intuitive visual examination of CNV has been an important trend in recent years [31]. For NGS, its accuracy is affected by noise sources caused by GC deviation, and uneven sequence reading coverage in the assembly process, and it cannot use the information of the B allele frequency [32]. Therefore, in recent years, the research of combining SNP array + NGS data for comprehensive CNV detection has also begun to appear [33, 34]. For example, in iCNV, the data of different platforms are firstly normalized and standardized, and then the hidden Markov model is used for joint segmentation [33]. Its accuracy of CNV detection was shown to be significantly higher than that of whole exome sequencing. However, these endeavors, such as SNP array data visualization and dual platform CNV detection method, are still in the exploratory stage. Whether their accuracy and cost performance are better than the existing methods in embryonic aneuploidy detection is worthy of further verification in future research.

Currently, our clinical decisions are based on the assumption that NGS can accurately diagnose mosaic embryos. However, if the SNP array is more accurate in the diagnosis of mosaic embryos, NGS may cause some mosaic embryos to be misdiagnosed as euploid and mistakenly transferred or as aneuploid and discarded, which may damage the clinical outcomes of patients, especially for those with a poor ovarian response. Our study supports the need to further explore its accuracy in mosaicism detection.

Importantly, the diagnosis of mosaicism (using either an SNP array or NGS) is not directly obtained by witnessing the chromosome karyotype of each cell in the biopsy sample, but is indirectly inferred from the occurrence of intermediate copy number signal on the platform profile (i.e. the “imbalance” of chromosome quantities between monosomy and disomy, or between disomy and trisomy) [35]. However, it is noteworthy that such a mosaicism detection method may not be able to distinguish some complementary errors. For example, the mixed samples of 40% monosomy cells and 60% trisomy cells cannot be distinguished from the mixed samples of 80% disomy and 20% trisomy, as both samples end up with the same result: 20% mosaicism. Therefore, if the same number of trisomy cells and monomer cells appear in a TE biopsy, the average result is going to be euploid. This greatly increases the complexity of the interpretation of the results, because a diagnosed “transferable” embryo may actually be abnormal. Moreover, an intermediate copy number signal can also occur due to other non-mosaicism causes, such as detection artifact/noise, amplification bias, pollution, mitotic status, variation of embryo biopsy technology, and laboratory conditions that also affect the intermediate copy number [35]. Although many reported studies support that such mosaicism detection platforms based on intermediate copy number, represented by NGS, can accurately and sensitively detect mosaic embryos, with more and more reports of healthy live births after mosaic embryo transfer, scholars have begun to question whether the current diagnostic criteria based on intermediate copy numbers can accurately reflect the true mosaic state of embryos and its value in predicting pregnancy outcomes. In fact, Paulson and Treff believe that “intermediate copy number” is a more accurate term representing such embryos compared to “mosaic” [35]. Therefore, although our results suggest the need to determine whether SNP or NGS is more accurate in mosaic detection, whether the “mosaicism” interpreted from the intermediate copy number can represent the mosaicism (two distinct cell types) in the biopsy is a larger problem that must be clarified. This supports the importance of the rigorous preclinical evaluation of current mosaic embryo diagnosis methods before clinical implementation.

Notably, 71.6% of the patients in this study received PGT-SR because of structural rearrangements (balanced translocations, inversions). These structural rearrangements typically appear in PGT-A results as partial aneuploidies (deletions/duplications) [36], so it is worth questioning whether they affect the accuracy of our study. However, the proportion of whole-chromosome aneuploid mosaic embryos and segmental chromosome aneuploid mosaic embryos in this study was similar (52.38% vs 46.67%), and the stratified analysis showed that the consistency between the NGS and SNP arrays was poor regardless of the type of mosaicism (mosaicism for whole chromosomal or segmental aneuploidy, Table 2). Therefore, we believe that although the PGT-SR cycles account for a large proportion of the mosaic embryos in all included cycles, it does not affect the results of this study. In patients with balanced translocation, the two balanced translocation chromosomes and their normal homologues of germ cells generally form a quadrivalent structure during meiosis, which theoretically can form at least 18 types of gametes. However, it is unclear whether this special meiotic chromosome separation process has an effect on the occurrence of embryonic mosaicism. In fact, Malmgren et al. used single cell CGH analysis to detect 94 blastomeres from 28 human cleavage embryos, and found that 100% of the cleavage embryos were mosaic, or even chaotic [37], which was higher than the 75% mosaicism rate previously reported in normal IVF cleavage embryos [38, 39]. At present, there is no research assessing whether a difference in the rate of mosaicism also exists in blastocysts between patients with or without chromosomal rearrangement, but it is an interesting question worth exploring in the future.

The major strength of this study was that in both tests, MDA was used for whole-genome amplification, and the biopsy and detection were carried out by the same laboratory and the same group of professionals at our center, avoiding the deviations caused by different amplification methods, differences in personnel experience levels, and changes in laboratory conditions. Notably, due to the low initial DNA amount, two biopsies were often required for two PGTs in the past. However, in 2017, Gleicher et al. established a mathematical modeling method to show that single-point biopsy of six cells in a 300-cell trophectoderm cannot accurately reflect the multiplication of embryo mathematically, and at least 27 cells are required to achieve the minimum diagnostic predictability [40]. In 2020, Sachdev et al. re-biopsied 10 aneuploid embryos, four aneuploid embryos, and 18 mosaic embryos previously diagnosed using NGS, and verified the results using NGS [41]. They found that the reproducibility of the NGS results was higher than 95% in embryos previously identified as aneuploid and euploid, but low (35.2%) in embryos previously identified as mosaic, indicating an uneven distribution of abnormal cells in mosaic blastocysts [41]. Therefore, biopsy of two different trophectoderm regions may yield inconsistent results, especially for mosaic embryos. However, owing to the amplification of the DNA template using whole-genome amplification (WGA), we could carry out two tests in the same biopsy sample, avoiding the interference caused by two trophectoderm biopsies, which is another noteworthy strength of our study.

The major limitation of this study is that the sample size was small. Moreover, only a small number of FET cycles were performed, and the results of prenatal diagnosis and chromosomal detection of abortion tissues were lacking. Besides, we only evaluated the consistency of the two platforms based on the consistency of the chromosome arms in which the mosaicism is located, regardless of whether the results of segmental chromosome abnormalities were consistent. However, even in this rough assessment, our study showed a very low consistency between the two detection platforms, indicating the significance of our study. Additionally, due to the retrospective nature of this study, a third technique was not introduced to confirm which platform’s test results are correct. In the future, a third aneuploid screening platform could be introduced to detect embryos with conflicting SNP array and NGS results. For example, qPCR can be considered, as described by Tan et al. [22] In addition, complete information from prenatal diagnosis and abortion tissue chromosome test results are required for verification, so as to accurately reflect the efficiency of the two platforms in diagnosing mosaic embryos.

## Conclusions

With the improvement in the sensitivity and resolution of PGT-A platforms, the detection rate of mosaic embryos is increasing. However, these improvements have also complicated clinical decision-making: discarding mosaic embryos may result in the wastage of many embryos with the potential to achieve a live birth. However, in comparison with euploid embryo transfer, the pregnancy outcomes of mosaic embryo transfer are not ideal. Therefore, accurate evaluation of mosaic embryos is very important for improving the clinical outcomes of patients undergoing PGT-A, especially those with a poor prognosis. This study is the first to compare the detection ability of the two most commonly used PGT-A platforms, NGS and SNP array, for mosaic embryos. The results of this study preliminarily show that the consistency between the two detection platforms for the diagnosis of mosaic embryos is low, and some of the embryos diagnosed as mosaic using SNP array are detected as aneuploid or aneuploid using NGS. Our results raise relevant questions about the accuracy of the two platforms in mosaicism detection, especially for NGS, which is becoming the mainstream testing platform in the field of PGT-A. Thus, larger and well-designed studies should be performed in the near future to verify the accuracy of the SNP array and NGS in the diagnosis of mosaic embryos, so as to facilitate more accurate clinical decision-making.

## Methods

### Study design

This retrospective study was performed using data from PGT-A or PGT-SR cycles conducted at the reproductive medicine center of the First Affiliated Hospital of Sun Yat-sen University from January 2018 to October 2020. We obtained the data for embryos detected as showing mosaicism with an SNP array, verified them via NGS detection, and then compared the two detection results.

### Trophectoderm biopsy and whole-genome amplification

We used conventional protocols for controlled ovulation hyperstimulation, intracytoplasmic sperm injection, and blastocyst culture [42, 43]. For fresh or vitrified thawed blastocysts, a non-contact laser was used for assisted hatching on the morning of the fifth or sixth day of fertilization. After 3–5 h, a biopsy of 5–10 trophectoderm cells was performed with the aid of the OcttaxShotTM laser system [44]. After biopsy, the blastocysts were vitrified using the Kitazato vitrification kit (Kitazato Biopharma Co., Ltd.). The biopsied trophectoderm sample was amplified using MDA for WGA. The MDA kit used was the REPLI-g Single Cell WGA kit (QIAGEN), and all operations were performed in strict accordance with the kit instructions. Finally, 50 μL of the MDA products were obtained from each embryo biopsy sample.

### SNP array and NGS

For each embryo, 4 μL of the corresponding MDA products were hybridized with the Human CytoSNP-12 chip (Illumina), which contains nearly 300,000 genetic markers. The hybridized chip was scanned using the iScan system (Illumina), and the data were analyzed using GenomeStudio software 2.0 (Illumina). We used median LogR deviation and median call rate to evaluate cytosnp-12 chip data. Median LogR deviation < 0.2 and median call rate > 0.98 were defined as the criteria for good data. The SNP array uses the log R ratio and the B allele frequency (BAF) of SNPs to determine the ploidy of chromosomes. When the BAF scatter of heterozygous SNP loci is not concentrated at 0.5, Log R > 0.2 or < − 0.2 indicates the occurrence of mosaicism [45].

For embryos diagnosed as mosaic using the SNP array, another 4 μL of the corresponding MDA products were sampled and tested with NGS. The Veriseq PGS-MiSeq kit (Illumina) was used to construct the library of MDA products, and the samples were sequenced on the MiSeq Sequencer (Illumina) using the MiSeq Reagent Kit V3 (Illumina). Secondary analysis was performed using MiSeq Reporter software (Illumina). For each sample tested with NGS platform, no less than 0.7 million single-end (SE) 36 bp reads are generated, and Map_Ratio, Duplicate, GC_Content, and SD was statistically calculated for data quality control for data quality control. Map_Ratio < 80% indicates that there may be laboratory pollution of non-human molecules, or the sample to be tested has not been successfully obtained, or the sequencing instrument fails. Duplicate reflects the quality of amplified products and libraries and is closely related to library diversity. GC < 39% or GC > 45% represents the possible genome-wide amplification imbalance. Segmental duplication, deletion above 4 MB and whole chromosomal aneuploidy can be detected only when SD < 3.5. NGS evaluates chromosome ploidy by detecting copy number variation (CNV) values. That is, when the CNV value is between 1.20–1.80 and 2.20–2.80, the embryo is diagnosed as mosaic, when the CNV value is < 1.20 or > 2.80, the embryo is marked as pure aneuploidy, and when the CNV value ranges from 1.80–2.20, the embryo is diagnosed as euploidy [43].

Due to the difference in the ability of the two methods to detect segmental chromosomal aneuploidy (the resolution of NGS is much higher, thus detects significantly more CNVs), the consistency of the two platforms was evaluated by judging the consistency of the chromosome arms in which the mosaicism is located, regardless of whether the results for segmental chromosome abnormalities are consistent. Complete concordance was concluded when the results obtained with NGS and the SNP array showed complete agreement for the chromosome arms involved in mosaicism. Partial concordance was concluded when the results obtained with the two platforms showed partial agreement for the chromosome arms involved in mosaicism. Partial concordance was concluded when the chromosome arms involved in mosaicism detected using the two platforms were partially but not completely consistent. If NGS detected additional chromosome arms involved in mosaicism while detecting all of those present in the SNP array, it was also concluded as partial concordance. Discordance was concluded when the results obtained with the two platforms identified completely different chromosome arms involved in mosaicism or when an embryo diagnosed as showing mosaicism using the SNP array showed a euploid status or aneuploidy on NGS.

It should be noted that even mosaic deletion and mosaic duplication which occurring on the same chromosomal arm belonged to different types of mosaicism, which was also applicable to mosaic monosomy and mosaic trisomy of the same chromosome. Even on the same chromosome, mosaicism occurring on the whole chromosome and mosaicism occurring only on the chromosome arm belonged to different types of mosaicism.

### Frozen-thawed embryo transfer

Blastocysts diagnosed as euploid or showing low-level (≤50%) mosaicism using NGS were defined as transferable embryos, and those with high embryo quality (on the basis of the Blastocyst score proposed by Gardner and Schoolcraft in 2000) were given priority for FET [46]. Blastocyst thawing was conducted with the thawing kit (Kitazato BioPharma Co. Ltd., Japan). The patient underwent a natural cycle or hormone replacement treatment cycle for endometrial preparation, and single-blastocyst transplantation was performed on the fifth day after ovulation or the sixth day after endometrial transformation.

On the 14th day after embryo transfer, blood levels of human chorionic gonadotropin were examined to determine whether pregnancy had occurred. After 4–6 weeks of embryo transfer, if the intrauterine gestational sac and fetal heartbeat were observed, clinical pregnancy was confirmed. Live birth was defined as 28 weeks of gestation with at least one surviving newborn. Miscarriage that occurred before 12 weeks of pregnancy was defined as early miscarriage, and miscarriage that occurred after 12 weeks to less than 28 weeks of pregnancy was defined as late miscarriage.

### Statistical analysis

Continuous variables were expressed as mean ± SD. The Student’s t test was used to compare continuous variables with a normal distribution, and Mann–Whitney U test was used to compare continuous variables without a normal distribution. The count data were expressed as n (%) and analyzed using Pearson’s chi-square test. When any expected value was less than 5, a Fisher’s exact test was used. This study used IBM SPSS 23.0 software (IBM Corp., Armonk, NY, USA) for data analysis. *P* < 0.05 was considered to indicate statistical significance.

## Supplementary Information

**Additional file 1: Table S1.** The detailed SNP array and NGS results of the 105 alleged mosaic blastocysts diagnosed by SNP array.

## Data Availability

The dataset supporting the conclusions of this article is included within the article and its additional file.

## Abbreviations

PGT-A: Preimplantation genetic testing for aneuploidies
aCGH: Array-based comparative genomic hybridization
SNP: Single-nucleotide polymorphism
NGS: Next-generation sequencing
CCS: Comprehensive chromosome screening
FISH: Fluorescence in situ hybridization
qPCR: Quantitative polymerase chain reaction
MDA: Multiple displacement amplification
WGA: Whole-genome amplification
FET: Frozen-thawed embryo transfer
BAF: B allele frequency
