# Supplementary material for: The inconsistency between two major aneuploidy-screening platforms—single-nucleotide polymorphism array and next-generation sequencing—in the detection of embryo mosaicism
Source: BMC Genomics. 2022 Jan 18;23:62. doi: 10.1186/s12864-022-08294-1 (PMC8764859; doi:10.1186/s12864-022-08294-1)
Supplement: Supplementary file 1 — Additional file 1: Table S1. The detailed SNP array and NGS results of the 105 alleged mosaic blastocysts diagnosed by SNP array. [file 12864_2022_8294_MOESM1_ESM.docx]

**Table S1. The detailed SNP array and NGS results of the 105 alleged mosaic blastocysts diagnosed by SNP array**

| Blastcyst No. | Indication | SNP array | NGS | Transferable  Embryo |
| --- | --- | --- | --- | --- |
| Completely concordant | | | | |
| 1 | Recurrent miscarriage | -mos(6),-mos(21),-mos(X) | -mos(6)(47%),-mos(21)(42%), -mos(X)(59%) | No |
| 2 | Recurrent miscarriage,advanced maternal age | +mos(16) | +mos(16)(68%) | No |
| 3 | Recurrent miscarriage | -mos(11) | -mos(11)(34%) | Yes |
| 4 | Recurrent miscarriage | -mos(13) | -mos(13)(63%) | No |
| 5 | Recurrent miscarriage | -mos(4) | -mos(4)(38%) | Yes |
| 6 | Recurrent miscarriage | -mos(2q) | -mos(2q)(39%) | Yes |
| 7 | Recurrent miscarriage | -mos(20) | -mos(20)(32%) | Yes |
| 8 | 46,XY,t(7;22)(q36;q11.2) | -mos(2)(q32.1) | -mos(2)(q32.1)(50%) | Yes |
| 9 | 46,XX,t(3;5)(q27;q33.3) | +mos(15) | +mos(15)(48%) | Yes |
| 10 | 46,XX,t(10;21)(q24.1;q22.3) | -mos(4)(q28.2) | -mos(4)(q28.2)(46%) | Yes |
| 11 | 46,XY,t(1;19)(q11;q11) | -mos(4)(q34.1) | -mos(4)(q34.1)(27%) | Yes |
| 12 | 46,XX,t(4;15)(q25;q25) | -mos(6)(p22.3) | -mos(6)(p22.3)(38%) | Yes |
| 13 | 46,XY,t(11;12)(q13;p12) | -mos(4) | -mos(4)(37%) | Yes |
| 14 | 46,XX,t(11;22)(q23.3;q11.2) | +mos(6)(q22.1) | +mos(6)(q22.1)(54%) | No |
| 15 | 46,XX,t(11;22)(q23.3;q11.2) | +mos(6),+mos(8) | +mos(6)(45%),+mos(8)(40%) | Yes |
| 16 | 46,XY,t(10;18)(p13;q21.1) | -mos(7) | -mos(7)(44%) | Yes |
| 17 | 46,XY,inv(9)(p11q13),t(18;19)(p11;p13) | -mos(2) | -mos(2)(48%) | Yes |
| 18 | 46,XY,t(12;15)(q24.3;q23) | -mos(7) | -mos(7)(39%) | Yes |
| 19 | 46,XY,t(4;14)(q35;q24.1) | -mos(9q) | -mos(9q)(41%) | Yes |
| 20 | :46,XY,t(6;8)(q21;q24.22) | -mos(5) | -mos(5)(25%) | Yes |
| 21 | 46,XX,t(9;21)(q22.3;q22.1) | -mos(1) | -mos(1)(40%) | Yes |
| 22 | 46,XY,t(8;13)(p22;q14.1) | -mos(18) | -mos(18)(61%) | No |
| 23 | 46,XY,t(8;13)(p22;q14.1) | -mos(9) | -mos(9)(38%) | Yes |
| 24 | 46,XY,t(5;11)(q35.3;p15.3) | -mos(16q) | -mos(16q)(38%) | Yes |
| 25 | 46,XY,t(5;11)(q35.3;p15.3) | -mos(7p) | -mos(7p)(62%) | No |
| 26 | 46,XX,t(16;19)(p13.3;p13.2) | +mos(10) | +mos(10)(66%) | No |
| 27 | 46,XY,t(8;21)(q21.2;p11.2) | -mos(7)(q31.1) | -mos(7)(q31.1)(43%) | Yes |
| 28 | 46,XY,t(5;8)(q31;p22) | +mos(8) | +mos(8)(44%) | Yes |
| 29 | 46,XX,inv(5)(q22q35.3) | -mos(4)(q26) | -mos(4)(q26)(56%) | No |
| 30 | 46,XY,t(2;10)(q13;p11.2) | -mos(1)(p35.3) | -mos(1)(p35.3)(46%) | Yes |
| 31 | 46,XX,t(4;14)(q33;q13) | -mos(17q) | -mos(17q)(40%) | Yes |
| 32 | 46,XX,t(10;22)(q23;q13) | -mos(1q) | -mos(1q)(39%) | Yes |
| 33 | 46,XY,t(4;7)(q21.3;q21.2) | -mos(17)(p12) | -mos(17)(p12)(46%) | Yes |
| 34 | 46,XX,t(2;3)(p23;q23) | -mos(9)(p21.1) | -mos(9)(p21.1)(44%) | Yes |
| 35 | 46,XX,t(10;20)(p11.2;p12) | -mos(13)(q14.11) | -mos(13)(q14.11)(59%) | No |
| 36 | 46,XY,t(8;14)(p21.1;q32.1) | +mos(3)? | -mos(3)(34%) | Yes |
| 37 | 46,XY,t(8;9)(q13;q21) | +mos(2) | +mos(2)(32%) | Yes |
| 38 | 46,XX,t(7;15)(q31.33;q26.3) | -mos(11)(q13.1) | -mos(11)(q13.1)(29%) | Yes |
| 39 | 46,X,Yqh-,t(2;5)(q14.2;q34) | -mos(9) | -mos(9)(42%) | Yes |
| 40 | Recurrent miscarriage,advanced maternal age | -mos(14)(q21.3) | -mos(14)(q21.2)(50%) | Yes |
| 41 | Recurrent miscarriage | -mos(10p) | -mos(10)(p15.3p11.1)(39%) | Yes |
| 42 | 46,XY,t(1;20)(p13.3;p13) | -mos(14)(q21.3) | -mos(14)(q21.2)(39%) | Yes |
| 43 | 46,XX,t(15;22)(q26.1;q12.2) | -mos(13)(q14→q32) | -mos(13)(q31.3)(44%) | Yes |
| 44 | 46,XX,t(15;22)(q26.1;q12.2) | -mos(7)(q11.23) | -mos(7)(q22.3)(63%) | No |
| 45 | 46,XX,t(12:14)(p12;q21) | -mos(17)(q24) | -mos(17)(q22)(41%) | Yes |
| 46 | 46,XY,t(1;12)(q25.1;q24.1) | -mos(3)(p24.3) | -mos(3)(p14.2)(38%) | Yes |
| 47 | Recurrent miscarriage | -mos(4)(q13.3) | -mos(4)(q21.1)(41%) | No |
| 48 | 46,XX,t(5;17)(p10;q10) | -mos(12)(q23.2) | -mos(12)(q23.3)(41%) | Yes |
| 49 | 46,XX,t(8;18)(q22.1;q21.1) | -mos(1)(p34) | -mos(1)(p34.1)(30%) | Yes |
| 50 | Recurrent miscarriage, recurrent implantation failure | -mos(2)(q22.3),-mos(4)(q22.1) | -mos(2)(q22.3)(54%),-mos(4)(q25)(38%) | No |
| Partially concordant | | | | |
| 51 | Recurrent miscarriage | -mos(4)(q12) | -mos(4)(q12)(52%), +mos(21)(59%) | No |
| 52 | Recurrent miscarriage | -mos(4)(q12) | -mos(4)(q12)(59%),+mos(7)(40%),+mos(17)(44%) | No |
| 53 | Recurrent miscarriage | -mos(16) | -mos(12)(33%),-mos(16)(41%),-mos(21)(34%) | Yes |
| 54 | 46,XX,t(2;11)(p21;q23.3)[8]/46,XX[22] | -mos(8)(q22.3) | -mos(8)(q22.3)(52%),+mos(15)(45%) | No |
| 55 | 46,XX,t(11;22)(q23.3;q11.2) | -mos(6) | -mos(3)(43%),-mos(6)(31%),-mos(10)(41%) | Yes |
| 56 | 46,XX,t(5;20)(q11.2;p11.2) | -mos(5)(q35.1),-mos(22)(q12.1) | -mos(5)(q35.1)(49%),-mos(22)(q12.1)(33%),-mos(X)(37%) | Yes |
| 57 | 46,XY,t(14;18)(q11.2;p11.2) | -mos(2) | -mos(2)(31%),+mos(20)(33%) | Yes |
| 58 | 46,XX,t(11;22)(q23.3;q11.2) | -mos(4) | +mos(3)(28%),-mos(4)(27%) | Yes |
| 59 | 46,XY,t(9,22)(q13;q11.2) | -mos(6)(q24.3) | +mos(3)(39%),-mos (6)(q24.3)(40%),+mos(19)(47%) | Yes |
| 60 | Recurrent miscarriage | +mos(3),-mos(14) | -mos(14)(38%) | Yes |
| 61 | Recurrent miscarriage | -mos(1),-mos(18) | -mos(18)(38%) | Yes |
| 62 | Recurrent miscarriage | +mos(6)(p22),+mos(11)(p15.1) | +mos(11)(p15.1)(53%) | No |
| 63 | Recurrent miscarriage | -mos(3),mos(9),mos(12),mos(14),mos(18) | -mos(3)(56%),+mos(5)(40%),+mos(15)(51%),-mos(16)(55%) | No |
| 64 | 46,XY,t(5:11)(q35.3;p15.3) | +mos(1),-mos(6) | -mos(6)(56%) | No |
| 65 | 46,XX,t(15;20)(q11.2;q11.2) | -mos(16),-mos(18),-mos(22) | +mos(15)(q13.3)(50%),-mos(18)(36%),-mos(22)(42%) | Yes |
| 66 | 46,XY,t(7;11)(q31.2;q23.3) | -mos(13),-mos(18) | -mos(18)(41%) | Yes |
| 67 | 46,XX,t(12;14)(p12;q21) | -mos(4)(p15.2),-mos(17),-mos(X) | -mos(4)(39%),+mos(8)(40%),-mos(9)(36%),-mos(X)(79%) | No |
| 68 | 46,XY,t(8;21)(q21.2;p11.2) | -mos(11q) | -mos(11)(q13.5)(57%),-mos(15)(40%) | No |
| 69 | 46,XY,t(4;7)(q21.3;q21.2) | +mos(7p),-mos(15)(q23),-mos(20)(q13.12) | -mos(20p)(33%),-mos(20)(q12)(42%) | Yes |
| Disconcordant | | | | |
| Detected as completely different types of mosaicisms by NGS | | | | |
| 70 | 45,XO[88]/47,XXX,[12] | +2(mos)? | -mos(7)(q33)(53%) | No |
| 71 | Recurrent miscarriage | -mos(22) | +mos(16)(p12.2-q21)(43%) | Yes |
| 72 | Recurrent miscarriage | mos(12) | -mos(17)(56%) | No |
| 73 | Recurrent implantation failure | +mos(4),+mos(7),+mos(15) | -mos(18)(38%),-mos(21)(48%) | Yes |
| 74 | 46,XY,t(2;20)(q35;q12) | -mos(X) | +mos(4)(q22.3)(50%),+mos(13)(q14.11q21.1)(55%) | No |
| 75 | 46,XX,der(3)t(3;9)(p21.1;q34.1),der(9)t(3;9)inv(9)(q33;q34.3) | +mos(15)(q21.3) | -mos(15)(q22.2)(40%) | Yes |
| 76 | 46,XX,t(3;19)(p23;q13) | +mos(14)(q22.2) | -mos(14)(q21.2)(35%) | Yes |
| 77 | 46,XY,t(1;12)(q25.1;q24.1) | -mos(9) | -mos(9)(q21.31)(39%) | Yes |
| 78 | 46,XX,t(5;8)(q11.2;p23.1) | -mos(13)(q14) | -mos(13)(30%) | Yes |
| 79 | 46,XY,t(6;7)(q23.3;q11.23),inv(9)(p12q13) | -mos(7)(q21.11) | +mos(5)(q34)(60%),-mos(7)(38%),+mos(13)(q14.2)(44%) | No |
| 80 | Recurrent miscarriage | +mos(6),+mos(11) | +mos(6)(q11.1)(32%) | Yes |
| Detected as euploidy by NGS | | | | |
| 81 | Recurrent miscarriage | -mos(4) | No chromosomal aneuploidy abnormalities (over 4Mb) were observed | Yes |
| 82 | 46,XX,t(1;11)(p36.1;q23) | +mos(4) | No chromosomal aneuploidy abnormalities (over 4Mb) were observed | Yes |
| 83 | Recurrent miscarriage | mos(1),mos(3) | No chromosomal aneuploidy abnormalities (over 4Mb) were observed | Yes |
| 84 | Recurrent miscarriage | mos(11) | No chromosomal aneuploidy abnormalities (over 4Mb) were observed | Yes |
| 85 | 46,XY,t(7:9)(p22;q22.1) | +mos(7) | No chromosomal aneuploidy abnormalities (over 4Mb) were observed | Yes |
| 86 | 46,XY,t(2;21)(p11.2;q11.2) | mos(5)? | No chromosomal aneuploidy abnormalities (over 4Mb) were observed | Yes |
| 87 | 46,XX,t(9;13)(p24;q13) | +mos(3) | No chromosomal aneuploidy abnormalities (over 4Mb) were observed | Yes |
| 88 | 46,XX,t(2;8)(q31;q24.3) | +mos(2)(p24)? | No chromosomal aneuploidy abnormalities (over 4Mb) were observed | Yes |
| 89 | 46,XY,t(17;19)(q21.3;q13.1) | -mos(5)(p15.1) | No chromosomal aneuploidy abnormalities (over 4Mb) were observed | Yes |
| 90 | 46,XX,t(4;16)(p15.2;q23) | +mos(2) | No chromosomal aneuploidy abnormalities (over 4Mb) were observed | Yes |
| 91 | 46,XY,t(9,22)(q13;q11.2) | -mos(7)(q11) | No chromosomal aneuploidy abnormalities (over 4Mb) were observed | Yes |
| Detected as aneuploidy by NGS | | | | |
| 92 | 46,XX,t(4;6)(p14;q13) | mos(10)(q25.3) | +(10)(q25.3) | No |
| 93 | Recurrent miscarriage,advanced maternal age | -mos(15) | -15 | No |
| 94 | 46,XY,t(4;7)(q25;p15.1) | +mos(22) | +22 | No |
| 95 | 46,XX,t(4;13)(q35;q22) | -mos(7p) | -(7p) | No |
| 96 | 46,XX,t(4;6)(p14;q13) | mos(10)(q25.3) | +(10)(q25.3) | No |
| 97 | 46,XX,t(4;6)(p14;q13) | mos(10)(q25.3) | +(10)(q25.3) | No |
| 98 | 46,XX,t(4;13)(q35;q22) | -mos(9)(p21.2) | -mos(9)(p21.3)(60%),+(9)(p21.3p13.1) | No |
| 99 | 46,XY,t(1;8)(q43;q21.3) | +mos(8)(q23.1) | +(8)(q22.2) | No |
| 100 | 46,XX,t(1;11)(p36.1;q23) | +mos(15)(q11.1q21.2),-mos(15)(q21.2) | -(15)(q21.2) | No |
| 101 | Recurrent miscarriage | -mos(8)(q21.13) | +mos(3)(p12.1)(55%), -(8)(q21.12q22.1), -mos(8)(q22.1)(48%) | No |
| 102 | 47,XX+8[19]/46,XX,[11] | mos(16)(p12.3) | -mos(15)(47%),+16,+mos(19)(45%),+mos(22)(45%) | No |
| 103 | 46,XX,t(3;5)(q27;q33.3) | +mos(5) | +5,-(10)(p12.31p11.23),+19(q11q13.2) | No |
| 104 | 46,XY,t(5;7)(q23.2;q36) | -mos(3),+mos(18q) | -mos(3)(60%),-(11)(q14.1q22.1),+mos(12)(54%),+(17q),-mos(X)(43%) | No |
| 105 | 46,Y,t(X;6)(q22.2;q12) | -mos(4)(q13.1)? | +(7)(q21.1-q21.3),-mos(11)(q14.1)(45%) | No |

SNP: Single Nucleotide Polymorphism; NGS: Next Generation Sequencing.
